# Current News

**Published:** 1889-06

**Authors:** 


### Current News.

The Boston meeting was a decided success.

Are you going to Newport to attend the meeting of the American Medical the last week in June?

The Dental section of the American Medical Association promises to be of greater interest this year than formerly.

All Dentists who desire to see dentistry recognized as a medical specialty should attend the regular annual meeting of the American Medical Association to be held at Newport, R. I., June 25th, 1889.

### NEW JERSEY STATE SOCIETY.

The nineteenth annual session of the New Jersey State Dental Society will be held at the West End Hotel, Asbury Park, N. J., commencing Wednesday, July 17th, and continuing the 18th and 19th. Prominent dentists from throughout the country will read interesting papers, and the clinics will be more than usually instructive. Everything new and useful in operative or mechanical dentistry will be exhibited, preparations having been made for plenty of space. Low hotel rates will prevail.

CHARLES A. MEEKER, D. D. S.,
*Secretary.*

---

IMPLANTATION DATA DESIRED.—In order to obtain and place on record a consensus of professional experience and judgment in regard to *Dental Implantation*, it is desired that dentists in this and foreign countries will kindly submit to the Chairman of Section VI, of the American Dental Association, facts called for under the following inquiries:

1. Date of each operation, sex, age, temperment and physical condition of the patient.
2. Description of the implanted tooth; date of its extraction; condition of the peridental membrane; preparation of the root by trimming or shortening; filling material used in the root canal.
3. Antiseptics used in preserving the tooth for implantation, and during the operation; also the antiseptic washes prescribed after the operation.
4. Instruments employed in the preparation of the artificial socket, and the method of sterilizing the same.
5. Appliance used for retaining in place the implanted tooth, and the length of time employed.
6. Subsequent history of each case to date of report, or up to the time when last seen or heard from.
7. In cases which have failed, specify minutely the causes of failure, together with an exact description of the appearance of the implanted tooth after extraction.
8. Total number of teeth implanted; number deemed successful, and the number known to have failed.

It is earnestly requested that facts of the kind above indicated shall be sent before the first of July next, to Dr. H. A. Smith, No. 128 Garfield Place, Cincinnati, Ohio.

H. A. SMITH.

Cincinnati, Ohio, May 20, 1889.

